# Lottery or Triage? Controlled Experimental Evidence from the COVID-19 Pandemic on Public Preferences for Allocation of Scarce Medical Resources

**DOI:** 10.1177/0272989X251367777

**Published:** 2025-09-27

**Authors:** Rhys Llewellyn Thomas, Laurence SJ Roope, Raymond Duch, Thomas Robinson, Alexei Zakharov, Philip Clarke

**Affiliations:** Health Economics Research Centre, Nuffield Department of Population Health, University of Oxford, Oxford, UK; Health Economics Research Centre, Nuffield Department of Population Health, University of Oxford, Oxford, UK; NIHR Oxford Biomedical Research Centre, John Radcliffe Hospital, University of Oxford, Oxford, UK; Nuffield College, University of Oxford, Oxford, UK; London School of Economics and Political Science, London, UK; Harris School of Public Policy, University of Chicago, Chicago, IL, USA; Faculty of Economics, Higher School of Economics, Moscow, Russia; Health Economics Research Centre, Nuffield Department of Population Health, University of Oxford, Oxford, UK

**Keywords:** vaccine allocation, COVID-19, lottery allocation, public preferences, cross-country comparision, survey experiment

## Abstract

**Background:**

Bioethicists have advocated lotteries to distribute scarce health care resources, highlighting the benefits that make them attractive amid growing health care challenges. During the COVID-19 pandemic, lotteries were used to distribute vaccines within priority groups in some settings, notably in the United States. Nonetheless, limited evidence exists on public attitudes toward lotteries.

**Methods:**

To assess public support for vaccine allocation by lottery versus expert committee, we conducted a survey-based experiment during the pandemic. Between November 2020 and May 2021, data were collected from 15,380 respondents across 14 diverse countries. Respondents were randomly allocated (1:1) to 1 of 2 hypothetical scenarios involving COVID-19 vaccine allocation among nurses: 1) by lottery and 2) prioritization by a committee of expert physicians. The outcome was agreement on the appropriateness of the allocation mechanism on a scale ranging from 0 (*strongly disagree*) to 100 (*strongly agree*), with differences stratified by a range of covariates. Two-sided *t* tests were used to test for overall differences in mean agreement between lottery and expert committee.

**Findings:**

Mean agreement with lottery allocation was 37.25 (95% confidence interval [CI] 34.86–39.65), ranging from 21.1 (95% CI 15.07–27.13) in Chile to 62.33 (95% CI 54.45–70.21) in India. In every country, expert committee allocation received higher support, with mean agreement of 61.19 (95% CI: 60.04–62.35), varying from 51.25 in Chile to 69.77 in India. Greater agreement with lotteries was observed among males, higher-income individuals, those with lower education, and those identifying as politically right leaning.

**Conclusions:**

Despite arguments for lottery-based allocation of medical resources, we found low overall public support, albeit with substantial variation across countries. Successful implementation of lottery allocation will require targeted public engagement and clear communication of potential benefits.

**Highlights:**

- This study surveyed 15,380 respondents from 14 diverse countries during the COVID-19 pandemic, analyzing international agreement with the appropriateness of using lottery allocation for scarce health care resources.
- There was universal preference for allocating vaccines by expert committee rather than by lotteries, but there was significant variation in agreement between countries, indicating the need for region-specific policy approaches.
- Successful implementation of lottery allocation requires targeted public engagement and communication of their benefits, especially with groups less supportive of lotteries.

The use of lotteries to allocate resources has a long history.^1^ Lotteries have been used both to allocate public duties, such as military drafts in the United States, and to allocate wide-ranging goods, from hunting permits (United States) to secondary (high) school and medical school admissions in England and the Netherlands, respectively.^2–4^ While some have argued that such mechanisms can be inefficient from an allocative point of view (e.g., in the context of the military draft^5^), others have argued that lotteries are a fair and relatively low-cost way of allocating indivisible goods.^1^

In the case of scarce medical treatments, many have endorsed random selection, in the form of lotteries, as a fair basis of allocation,^6,7^ especially within priority categories, where choosing between similar patients can be challenging. This approach aligns with principles of justice by giving everyone an equal chance of receiving medical resources, regardless of their condition or prognosis, and thus prioritizes equality.^9^ Lotteries are also appealing because they are transparent, impartial, and inexpensive to administer. However, despite these advantages, lotteries also have notable drawbacks.^8^ In the context of medical treatment allocation, the most significant consideration is that random assignment may lead to suboptimal use of limited resources. While lotteries ensure equality, they are unable to guarantee that societal benefits are maximized.^9^

If the objective is to maximize the overall benefits of scarce treatments, then allocation of resources by medical triage may be a preferred alternative. Allocation through expert triage seeks to optimize outcomes, which may be lives saved or quality-adjusted life-years. Nevertheless, such approaches are not without criticism.^9,10^ They may be viewed as unfair or discriminatory and rely on the assumption that medical professionals can accurately determine the most beneficial use of resources.^10^ In reality, optimizing social benefits is challenging due to uncertainties in patient prognosis, variability in treatment responses, and the inherent limitations of medical knowledge. Triage systems can also be used to prioritize certain groups, such as essential workers, to maintain critical services.^8^ While this can be justified on practical grounds, it may also be seen as placing greater value on some lives over others. Conversely, prioritizing those worst off aligns with principles of protecting the most vulnerable but may not lead to the greatest overall health outcomes.^9^

Each allocation method presents distinct ethical implications and practical challenges.^9,10^ Choosing between a lottery system and medical triage ultimately involves a tradeoff between equality of opportunity and the maximization of benefits. The selection of an allocation strategy must therefore strike a balance between competing ethical principles and practical constraints, reflecting broader societal values and what is deemed an acceptable and fair approach.

In practice, the use of lotteries in the allocation of health care has been relatively limited. A rare example of lottery allocation in a medical context took place in Oregon, which allocated access to Medicaid insurance by lottery.^11^ However, it was during the initial phases of the COVID-19 pandemic, when there were limited quantities of health care interventions such as ventilators and therapeutic drugs, that the proposed use of lotteries as a mechanism for allocating scarce medical resources gained wider prominence. A widely cited article in the *New England Journal of Medicine*^12^ made 6 key recommendations for the allocation of scarce medical resources during the COVID-19 pandemic. These included prioritizing the maximization of benefits and saving the greatest number of lives, giving precedence to frontline health care workers, adapting policies in response to emerging evidence, recognizing the contributions of research participants, avoiding first-come, first-served allocation, and ensuring that principles were applied consistently across all patients, regardless of diagnosis. To address the shortcomings of first-come, first-served systems and to promote fairness among patients with similar prognoses, the panel explicitly recommended the use of lotteries as a method of rationing. Their third recommendation argued that “for patients with similar prognoses, equality should be invoked and operationalized through random allocation, such as a lottery” and stated that random allocation is ethically preferable to first-come, first-served allocation. Subsequently, lottery allocation of COVID-19 vaccines was advocated not only in academic journals^13^ and by organizations such as Gavi^14^ but also in popular media including the *New York Times.*^15^ Lotteries were also incorporated in recommendations of the US National Academy of Sciences’ report on COVID-19 vaccine prioritization^16^ as part of a combined triage–lottery allocation system. The report advocated prioritization for vaccination based on 6 ethical and procedural principals (i.e., expert triage), concluding that high-risk health workers, first responders, people with significant comorbid conditions, and older adults in congregate or overcrowded settings should be in the first phase of vaccination. However, the report also stated that, “If the supply of vaccine is too limited to provide it to everyone in a particular priority group . . . the principle of equal concern can support random selection” (i.e., lottery). Furthermore, the report also discussed that lotteries have the benefit of balancing confounders (both observed and unobserved) across those that received the vaccine and those that did not, thereby creating a natural experiment to test vaccine effectiveness. The report also acknowledged that a weighted lottery (i.e., a lottery with increased chances of winning for certain groups) “could be used to fairly allocate the scarce supply of vaccine with certain groups receiving heightened priority.”

In practice, the use of lotteries during the COVID-19 pandemic was quite limited. The US state of Pennsylvania endorsed a weighted lottery system for allocation of scarce COVID-19 drugs,^8^ and lotteries were used in the early phases of the vaccine rollout.^17^

Ethical debates over the merits of lottery allocation of vaccines in the event of a pandemic date back more than a decade,^18–20^ and the issue was even explored in the 2011 film *Contagion*, in which a birthday lottery was used to prioritize access to the vaccine.^21,22^ Prior to the COVID-19 pandemic, a small number of studies provided evidence on public attitudes to random allocation of medical resources in hypothetical situations ranging from emergency ward accident care in Israel,^23^ to allocation of donor organs, hospital beds during an epidemic, and joint replacements in German-speaking Switzerland.^24^ These studies suggest a limited degree of support among the public for lottery allocation, with less support than for allocation on the basis of need.^23–25^ They also indicate less support for lotteries among the public than among bioethicists.^24^ Grover et al.,^25^ for instance, reported the results of a survey of 515 individuals who were asked to rank order 8 different ethical positions with respect to the allocation of scarce resources. Here, “saving the most lives” and allocating the “sickest first” were the most supported principles, with lotteries the least favored. Awad et al.^26^ found evidence from representative samples for 4 countries (*N* = 4,000) together with nonrepresentative (self-selected) samples from 20 countries (*N* = 7,599) that preferences for allocation of scarce medical resources are polarized, with large groups preferring either extensive triage or no triage (random allocation or first come, first served). One limitation of these studies is that respondents are asked their preferences for several different allocation methods within the same questionnaire or survey, and therefore there may be contamination effects when respondents are made aware of alternative allocation mechanisms.

In this study, we aim to add to understanding of public agreement with the appropriateness of using a lottery as an allocation mechanism in 2 ways. First, we combine scale with reliability by fielding harmonized representative surveys across a greater range of countries than previous studies. Second, we use randomization to compare agreement with lottery versus expert committee allocation in a way that avoids any possible contamination of responses (e.g., that could potentially arise by asking the same respondents first about triage and then about lottery, or vice versa). We conducted an experiment to elicit the extent of public agreement, globally, with the use of lottery allocation as a mechanism for prioritizing access to COVID-19 vaccines, compared with support for allocation by expert committee. The experiment was conducted from November 24, 2020, to May 16, 2021, which in most countries was in the early stages of the vaccine rollout. It was in this context that the experiment aimed to understand the preferences of the public for lottery allocation versus more traditional expert committee. The study is based on a largely representative sample of 15,380 respondents from 14 economically and culturally diverse countries, representing roughly half the world’s population, and sought preferences regarding COVID-19 vaccine allocation.

With many countries now reviewing the policy response to the COVID-19 pandemic, it is worth gaining insight into the public’s views on lottery allocation. This can inform future policies to allocate rationed health care resources, either in future pandemics or more generally.

## Methods

### Data

The experiment was conducted as part of the first wave of the Oxford COVID-19 Vaccine Preference and Opinion Survey (CANDOUR) study,^27^ which used online surveys of adults older than 18 years to gather responses on a range of topics related to public and private health preferences, attitudes, and opinions. The survey aimed to elicit preferences regarding various aspects of COVID-19 vaccine allocation. The full list of questions asked of respondents is available in the replication materials for Duch et al.^27^ Surveys were conducted in 14 geographically diverse countries: Australia, Brazil, Canada, Chile, China, Colombia, France, India, Italy, Russia, Spain, Uganda, the United Kingdom, and the United States. The surveys were completed between November 24, 2020, and January 14, 2021. A slightly modified survey was undertaken in Russia between April 8 and May 16, 2021.

In all but Chile, Uganda, and Russia, the respondents were obtained from the sampling firm Respondi’s active panel. Respondi maintain a panel of individuals who completed a double opt-in registration, a profile of basic information, and enough surveys to be considered active. While Respondi’s exact panel recruitment method is proprietary, they constantly recruit new panelists through online advertising, search engine marketing, affiliate partners, and Facebook, with some supplementation from computer-assisted telephone interviews. The Respondi participants were compensated for completing the survey. For these 11 countries, the modal incentive was $3.50 for a median length of interview of 25.53 min. In Chile and Uganda, the respondents were sampled using Facebook Ad Manager. In Russia, the survey was administered by the Online Market Intelligence organization, who use a standing panel of respondents.

### Sample and Survey

In all countries, apart from India and Uganda, we employed quota sampling to ensure that national samples matched the national demographic profile (India and Uganda are primarily samples of urban communities). Our quota strategy generated distributions that were intended to match the populations on age, education, gender, and region.

The survey was conducted according to the University of Oxford’s policy for human subjects research and approved by the University of Oxford Medical Sciences Interdivisional Research Ethics Committee (MS IDREC) (approval ID: R72328/RE001), with informed consent being obtained from each participant before commencement of the survey. The survey was translated from English into 6 languages: Chinese, French, Italian, Spanish, Portuguese, and Russian. A professional translation firm was used to translate the surveys initially, and these were then reviewed by native speakers, including members of the study team, to check the accuracy of the wording and that no important nuances had been lost. As an additional check, as is standard in multilanguage studies, they were then independently translated back to English and assessed by the research team for any discrepancies or important loss of nuance. Subjects in all countries were provided with identical descriptions of the general experimental rules and procedures, all of which are described in detail in the replication materials.^28^

Among the Respondi panelists invited to take our survey, the completion rate was 35.4%, averaged across all countries. For online panel samples this represents the percentage of those invited from the opt-in panel pool who either complete the survey or who are dropped from the survey because they are ineligible (typically because a quota has been filled).^29^ These completion rates compare favorably to, for example, random digit dial samples, although the denominator for online panels is those already opted in.^30,31^

As the detailed discussion in the Supplementary Materials to Duch et al.^27^ (section 3.1: *Sample*, pp. XX–XXX) indicates, the distributions of key sample demographics resemble those of their populations. On some demographics, for selected countries, we observe deviations from population distributions. Generally, the sample gender distributions match the population. Median incomes (individual and household) for the samples resemble those for the population and typically deviate no more than 20%. In most countries, the better educated were overrepresented. In some countries (Chile, China, Colombia, and Uganda), young respondents were overrepresented in the samples. To address sample imbalances on key demographics, we implemented poststratification weighting; see the Supplementary Materials in Dutch et al.^27^ (section 3.2: *Sample Quotas and Weighting*, pp. XXXI–XLVII) for a description of the raking procedure used for estimating the weights and a description of the distributions of key demographics for the pre- and postweighted samples.

### Nature of the Experiment and Analysis

Our experiment builds on the design of a series of experiments that elicited preferences for lottery allocation from Dutch university students.^32^ These experiments used hypothetical scenarios involving life and death decisions, for example, where there are 2 patients who will die without treatment but only 1 hospital bed available. The study found that 36% of students considered the allocation of medical treatment based on a coin flip to be fair. When asked which of the 2 patients should receive treatment, most students stated that they were equally deserving. However, only 17% chose to use a coin flip to allocate treatment, instead selecting 1 of the 2 patients whom they judged to be equally deserving. Overall, the results indicated relatively low levels of support for lottery allocation compared with a deliberative choice for 1 of the 2 patients. The CANDOUR survey used in this article builds on previous work by fielding a harmonized survey on representative samples from more countries, providing reliable evidence across diverse social and economic contexts. In addition, our study uses a salient scenario involving COVID-19 vaccinations during the initial rollout period of the vaccines.

**Figure 1:**
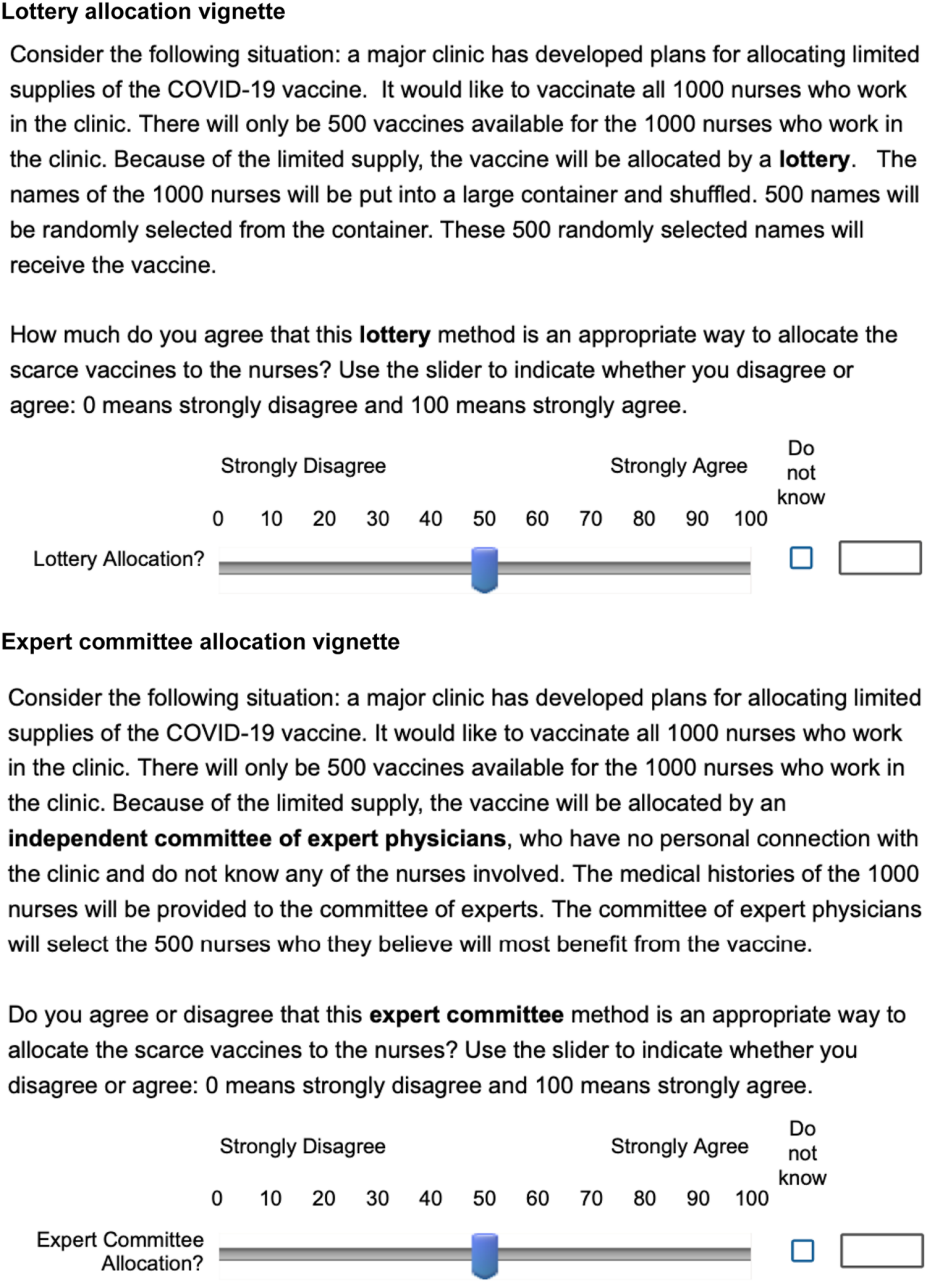
Wording of the 2 vignettes regarding the allocation of a COVID-19 vaccine in a hospital.

As shown in Figure 1, the experiment in the present study involved randomly assigning each participant to 1 of 2 alternative COVID-19 vaccine allocation scenarios in a hospital. The first scenario proposes a lottery to allocate the vaccine. A random half of respondents in each country were asked:

Consider the following situation: a major clinic has developed plans for allocating limited supplies of the COVID-19 vaccine. It would like to vaccinate all 1000 nurses who work in the clinic. There will only be 500 vaccines available for the 1000 nurses who work in the clinic. Because of the limited supply, the vaccine will be allocated by a **lottery**. The names of the 1000 nurses will be put into a large container and shuffled. 500 names will be randomly selected from the container. These 500 randomly selected names will receive the vaccine.

How much do you agree that this **lottery** method is an appropriate way to allocate the scarce vaccines to the nurses? Use the slider to indicate whether you disagree or agree: 0 means strongly disagree and 100 means strongly agree.

The alternative scenario proposes allocation by an independent committee of expert physicians (expert committee). Respondents in the other half of the sample in each country were asked the following:

Consider the following situation: a major clinic has developed plans for allocating limited supplies of the COVID-19 vaccine. It would like to vaccinate all 1000 nurses who work in the clinic. There will only be 500 vaccines available for the 1000 nurses who work in the clinic. Because of the limited supply, the vaccine will be allocated by an **independent committee of expert physicians**, who have no personal connection with the clinic and do not know any of the nurses involved. The medical histories of the 1000 nurses will be provided to the committee of experts. The committee of expert physicians will select the 500 nurses who they believe will most benefit from the vaccine.

Do you agree or disagree that this **expert committee** method is an appropriate way to allocate the scarce vaccines to the nurses? Use the slider to indicate whether you disagree or agree: 0 means strongly disagree and 100 means strongly agree.

Each participant only received 1 of these 2 vignettes and were blinded to any knowledge of alternative vignettes. The outcome of the experiment was agreement on a 0 to 100 scale, which respondents indicated using a slider.

### Sample Randomization and Statistical Methods

**Figure 2:**
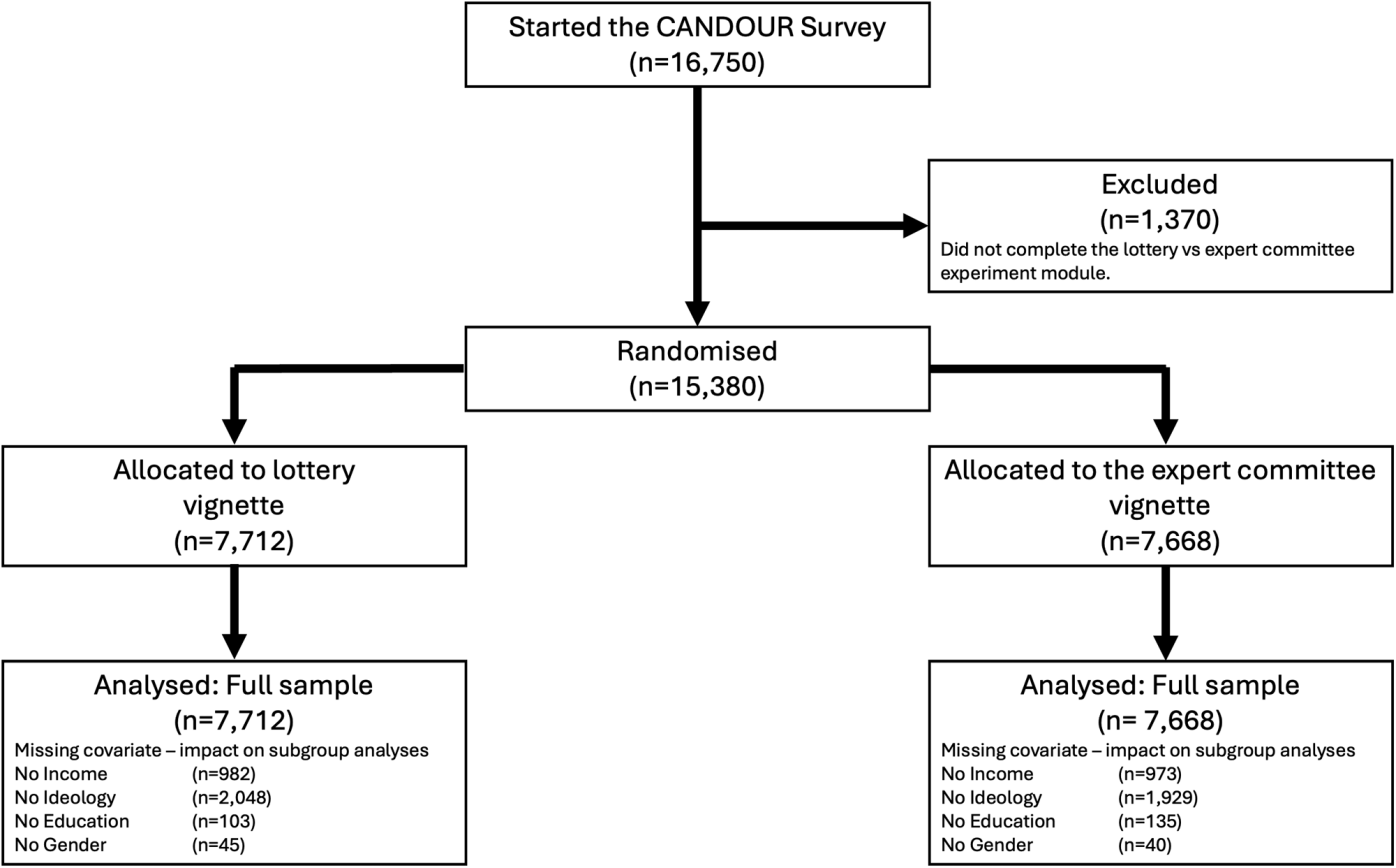
Study enrollment and missing covariate.

Figure 2 shows the randomization logic for subjects in this experiment. In total, 16,750 individuals participated in at least part of the survey. Of these, 15,380 participants were randomly allocated to an allocation vignette, with 7,712 receiving the lottery vignette and 7,668 receiving the expert committee vignette. Subjects’ agreement was recorded using a visual analog scale slider (ranging from 0 to 100), shown in Figure 1.

The primary outcomes of the study were the mean agreement for those given the lottery and the expert committee questions and the mean differences between agreement with the appropriateness of each allocation mechanism. Two-sided *t* tests were used to test for overall differences in mean agreement in the lottery and expert committee questions and for differences in mean agreement within individual countries and in subgroups defined by income, education, political ideology, and other demographic factors. In the supplementary materials, we also present the results of the multivariable regressions, where we assess the independent associations of a wide range of respondent characteristics, including their country, with agreement with the appropriateness of each allocation method.

## Results

The descriptive statistics across the 2 arms are displayed in Table 1. The *P* values for the *t* test (for continuous variables) and Pearson’s chi-squared test (for categorical variables) are presented in the right-most column of the table. Covariates are seen to be broadly balanced across the 2 arms, as expected with the sample size and the 1-1 randomization across the 2 arms. The only statistically significant difference is in education level (albeit not statistically significant if we use Bonferroni correction for multiple hypothesis), where the expert committee group has slightly more university-educated individuals and slightly fewer secondary-educated individuals, making them marginally more educated overall. This difference in groups has a very small impact on our main results (controlling for education category decreases the difference in mean agreement by 0.17%).

**Table 1:** Characteristics of the Participants

	Expert Committee	Lottery	*P-*Value Test for differences
Age, y	42.761 (15.728)	42.780 (15.827)	0.94
Age category, y			
18−39	3,657 (47.7%)	3,684 (47.8%)	0.74
40–60	2,532 (33.0%)	2,513 (32.6%)	
60+	1,471 (19.2%)	1,510 (19.6%)	
Missing/out of range	8 (0.1%)	5 (0.1%)	
Gender			
Female	3,683 (48.0%)	3,681 (47.7%)	0.78
Male	3,945 (51.4%)	3,986 (51.7%)	
Other	15 (0.2%)	21 (0.3%)	
Missing	25 (0.3%)	24 (0.3%)	
Political ideology			
Left	865 (11.3%)	847 (11.0%)	0.23
Center	3,573 (46.6%)	3,557 (46.1%)	
Right	1,301 (17.0%)	1,260 (16.3%)	
Missing	1,929 (25.2%)	2,048 (26.6%)	
Income category			
High	3,807 (51.2%)	3,908 (52.4%)	0.33
Low	2,888 (38.8%)	2,822 (37.8%)	
Missing	745 (10.0%)	729 (9.8%)	
Education			
Primary	882 (11.5%)	918 (11.9%)	0.017
Secondary	2,980 (38.9%)	3,129 (40.6%)	
University	3,671 (47.9%)	3,562 (46.2%)	
Missing	135 (1.8%)	103 (1.3%)	
Country			
Australia	588 (7.7%)	634 (8.2%)	0.44
Brazil	626 (8.2%)	670 (8.7%)	
Canada	509 (6.6%)	536 (7.0%)	
Chile	508 (6.6%)	484 (6.3%)	
China	617 (8.0%)	630 (8.2%)	
Colombia	615 (8.0%)	557 (7.2%)	
France	489 (6.4%)	502 (6.5%)	
India	580 (7.6%)	538 (7.0%)	
Italy	483 (6.3%)	489 (6.3%)	
Russia	583 (7.6%)	609 (7.9%)	
Spain	563 (7.3%)	510 (6.6%)	
Uganda	506 (6.6%)	521 (6.8%)	
United Kingdom	512 (6.7%)	534 (6.9%)	
United States	489 (6.4%)	498 (6.5%)	
Observations	7,668 (49.9%)	7,712 (50.1%)	

**Figure 3:**
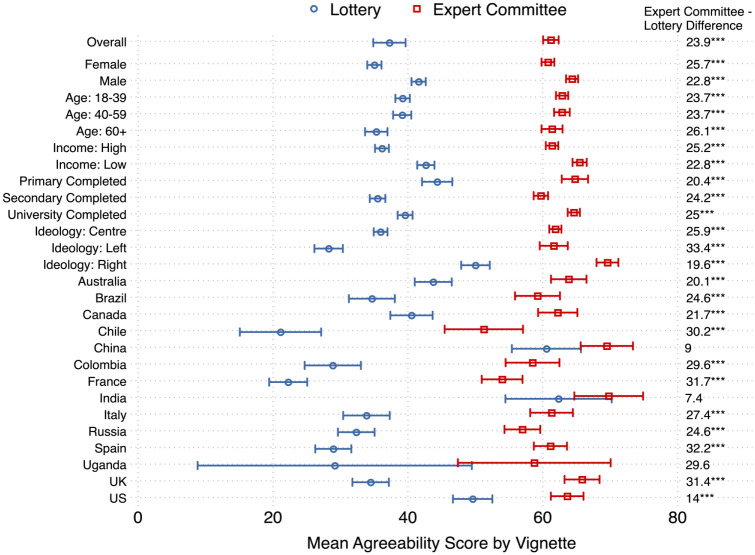
Preferences for expert committee allocation versus lottery allocation. The figure shows the mean agreement and 95% confidence intervals for the expert committee (red square) and lottery (blue circle) vignettes. Mean agreement is presented overall and by observable characteristics. Overall and country estimates use survey weights; estimates by respondent characteristics are unweighted. In addition, the figure displays the mean differences between expert committee and lottery allocation agreement, calculated using ordinary least squares, and the corresponding Bonferroni multiple hypothesis correction statistical significance. *P≤ 0.10; **P≤ 0.05; ***P≤ 0.01.

The results on average agreement for allocation by lottery versus expert committee are displayed in Table 2 and visually in Figure 3. The difference in mean score was 23.94 (95% confidence interval [CI] 21.27–26.61), meaning that allocation by expert committee was substantially more agreed with than allocation by lottery overall. However, there was reasonable variation in the mean agreement by respondent characteristics and by country. Average agreement with allocation by expert committee ranged from 51.25 (95% CI 45.44–57.06) in Chile to 69.77 (95% CI 64.67–74.87) in India. Agreement with lottery allocation varied much more markedly from 21.1 (15.07–27.13) in Chile to 62.33 (54.45–70.21) in India. In almost every country, there was a statistically significant difference in agreement between the appropriateness of lottery and expert committee allocation. Using Bonferroni’s multiple hypothesis correction, the only countries in which no statistically significant differences between expert committee and lottery agreeability were found was in China, India, and Uganda. In all countries, allocation by an expert committee received higher levels of mean agreement; however, the magnitude of this difference varied widely, ranging from 7.44 (−1.92 to 16.8) in India to 32.19 (28.57 to 35.81) in Spain. Across all observable subject characteristics, there was also higher agreement with expert committee than lottery allocation; however, the heterogeneity across these dimensions was less pronounced than across countries. There was greater agreement with lotteries among males, those on higher income, those with the very lowest levels of education, and those identifying with a right-leaning ideology.

**Table 2:** Preferences for Allocation by Lottery versus Expert Committee

	Expert Committee		Lottery		Expert Committee Mean – Lottery Mean (95% CI)
Characteristic	*n*	Mean (95% CI)	*n*	Mean (95% CI)	
Estimated using survey weights					
Overall	7,668	61.19 (60.04–62.35)	7,712	37.25 (34.86–39.65)	23.94 (21.27–26.61)
Country					
Australia	588	63.85 (61.21 to 66.48)	634	43.75 (41.01 to 46.5)	20.09 (16.3 to 23.89)
Brazil	626	59.21 (55.88 to 62.55)	670	34.65 (31.25 to 38.06)	24.56 (19.81 to 29.32)
Canada	509	62.21 (59.3 to 65.12)	536	40.52 (37.39 to 43.66)	21.69 (17.42 to 25.96)
Chile	508	51.25 (45.44 to 57.06)	484	21.1 (15.07 to 27.13)	30.15 (21.8 to 38.5)
China	617	69.49 (65.61 to 73.37)	630	60.54 (55.41 to 65.66)	8.96 (2.54 to 15.37)
Colombia	615	58.47 (54.48 to 62.47)	557	28.85 (24.69 to 33.02)	29.62 (23.86 to 35.38)
France	489	53.97 (50.94 to 56.99)	502	22.24 (19.42 to 25.06)	31.73 (27.61 to 35.85)
India	580	69.77 (64.67 to 74.87)	538	62.33 (54.45 to 70.21)	7.44 (−1.92 to 16.8)
Italy	483	61.3 (58.13 to 64.46)	489	33.85 (30.4 to 37.3)	27.45 (22.78 to 32.12)
Russia	583	56.97 (54.31 to 59.62)	609	32.33 (29.61 to 35.05)	24.63 (20.84 to 28.43)
Spain	563	61.13 (58.67 to 63.58)	510	28.93 (26.26 to 31.6)	32.19 (28.57 to 35.81)
Uganda	506	58.73 (47.4 to 70.06)	521	29.15 (8.83 to 49.48)	29.58 (6.38 to 52.79)
United Kingdom	512	65.82 (63.22 to 68.42)	534	34.47 (31.76 to 37.17)	31.35 (27.61 to 35.09)
United States	489	63.62 (61.2 to 66.03)	498	49.6 (46.67 to 52.52)	14.02 (10.24 to 17.8)
Estimated without survey weights					
Gender					
Female	3,683	60.76 (59.81 to 61.71)	3,681	35.02 (33.96 to 36.08)	25.74 (24.32 to 27.16)
Male	3,945	64.34 (63.45 to 65.24)	3,986	41.59 (40.53 to 42.65)	22.76 (21.37 to 24.14)
Age, y					
18–39	3,657	62.86 (61.96 to 63.76)	3,684	39.21 (38.13 to 40.28)	23.65 (22.25 to 25.05)
40–59	2,532	62.83 (61.67 to 63.99)	2,513	39.15 (37.8 to 40.49)	23.69 (21.91 to 25.46)
60+	1,471	61.37 (59.81 to 62.92)	1,510	35.3 (33.64 to 36.97)	26.07 (23.79 to 28.34)
Income					
Income: High	3,807	61.37 (60.44 to 62.29)	3,908	36.14 (35.11 to 37.17)	25.23 (23.84 to 26.61)
Income: Low	2,888	65.46 (64.41 to 66.51)	2,822	42.65 (41.37 to 43.93)	22.81 (21.16 to 24.46)
Education					
Primary completed	882	64.76 (62.81 to 66.71)	918	44.33 (42.09 to 46.58)	20.43 (17.46 to 23.4)
Secondary completed	2,980	59.71 (58.64 to 60.78)	3,129	35.49 (34.34 to 36.64)	24.22 (22.65 to 25.79)
University completed	3,671	64.59 (63.69 to 65.5)	3,562	39.58 (38.48 to 40.69)	25.01 (23.58 to 26.44)
Political ideology					
Center	3,573	61.86 (60.96 to 62.76)	3,557	35.95 (34.93 to 36.96)	25.91 (24.55 to 27.27)
Left	865	61.62 (59.55 to 63.7)	847	28.24 (26.13 to 30.35)	33.38 (30.43 to 36.34)
Right	1,301	69.59 (67.97 to 71.2)	1,260	50.01 (47.89 to 52.14)	19.57 (16.91 to 22.24)

The table shows the mean scores and 95% confidence intervals for the lottery and expert committee vignettes. Mean agreement is presented overall and by observable characteristics. In addition, the table displays the mean differences between agreement with the appropriateness of allocation by expert committee and lottery allocation and the corresponding 95% confidence interval, calculated using ordinary least squares.

**Figure 4:**
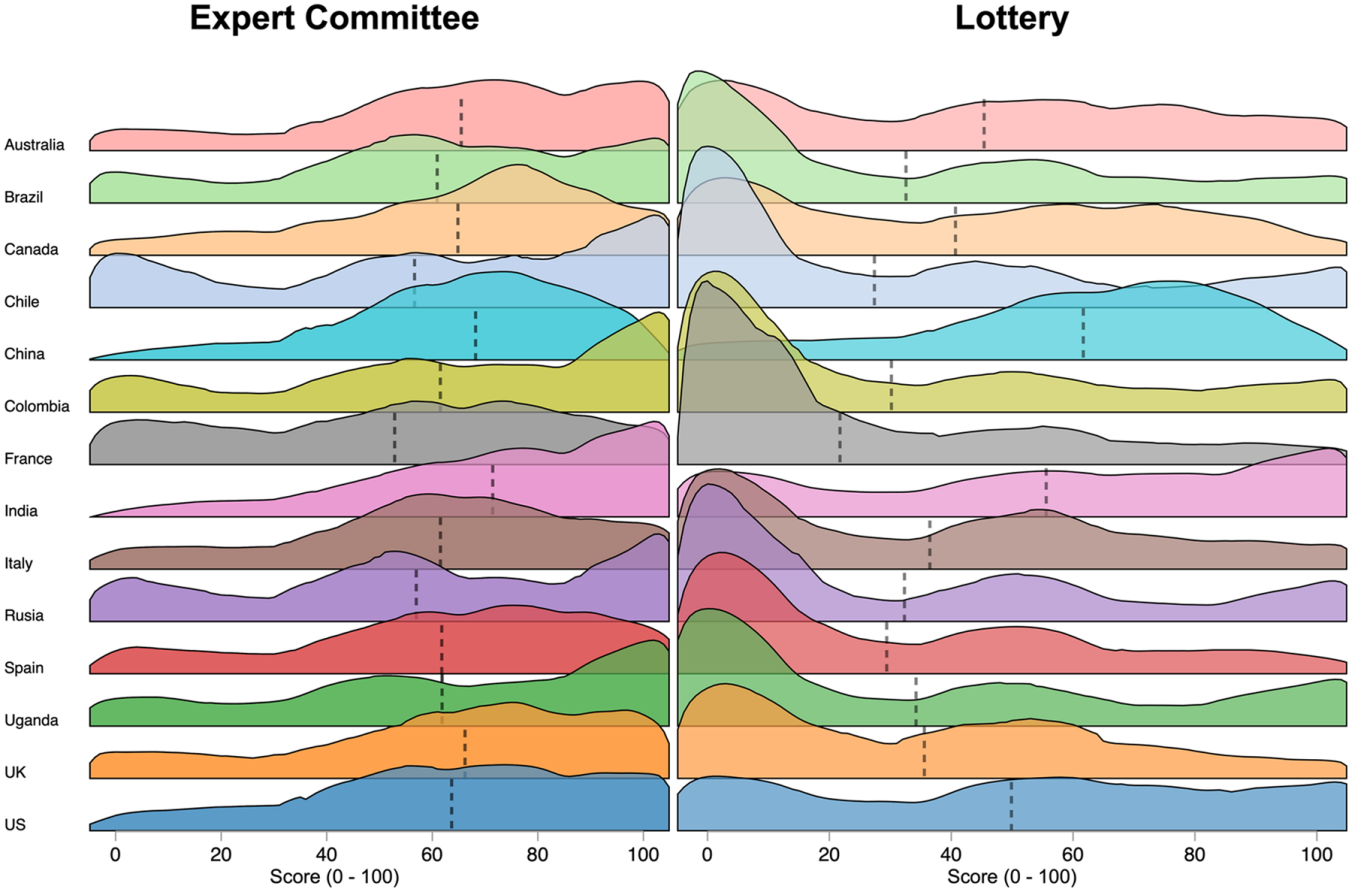
Distribution of agreement with the appropriateness of allocation by lottery and expert committee. Locally weighted regression (using density of the score as the dependent variable and score as the independent variable) is used to smooth the density plots. This is done separately for each country. The predictions of the locally weighted regressions are shown in these figures. Figures do not use sample weights. Vertical dashed lines represent the unweighted mean agreement with lottery or expert committee allocation for each country.

Figure 4 presents the distribution of scores by country. This figure shows that for most countries, notably excluding China and India, the highest density was at an agreement of 0 for lottery allocation. The distribution of scores in India and the United States was much more uniform, potentially suggesting greater diversity in views. The highest density for India was at an agreement of 100. The distribution of agreement in China suggests relatively strong agreement with lottery allocation of vaccines overall, with limited evidence of polarization in these views. It is also notable that agreement with lottery allocation was highest in India and China, which are not only the only 2 countries in the sample that lie fully within Asia but also those with by far the largest populations.

Figure 4 also shows the distribution of agreement for expert committee allocation. These figures suggest broad agreement with the appropriateness of expert committee allocation, with the peaks of the distributions being well to the right of 50 in all countries.

## Discussion

In the wake of the pandemic, a variety of reviews^33^ identified drug and medical care shortages as a key challenge for governments. These shortages go beyond times of crisis, such as pandemics. There are also ongoing worldwide shortages of many common medicines—particularly generic medicines and including a number of antibiotics^34^—that are likely to be a long-term challenge. While lottery allocation has been widely advocated as a rationing mechanism by bioethicists and endorsed by institutions such as the US National Academy of Sciences,^16^ it is rarely used for the allocation of treatments or vaccinations. This study explores public views on lottery allocation, using representative samples for a larger number of countries than in previous studies. Using a randomized approach that avoids any possibility of contamination, we assessed the level of public support for lottery allocation versus a more traditional approach of expert committee, in the context of allocation of COVID-19 vaccines.

Our study found relatively low overall agreement with lotteries being an appropriate means of allocating scarce medical resources but with substantial variation across countries, much more so than is the case with expert committee allocation. While it is not surprising that allocation by expert committee is more popular in all countries, the large variation both in agreement with lottery across countries and in the difference in agreement between expert committee and lottery is noteworthy. Ethical arguments for lottery allocation relate mainly to situations in which allocation is within groups of equal need. It is therefore of potential interest to policy makers that support for lottery allocation in such a context is likely to vary markedly by country context. Our results suggest that communicating the advantages of lotteries in such a circumstance may be more important, and more challenging, in countries such as France or Chile than in the United States, China, or India.

Interestingly, the United States was one of very few countries that incorporated a lottery into their allocation mechanism during the COVID-19 pandemic, both for medicines and vaccination appointments. For example, a hospital in a 35-hospital system in Pennsylvania, New York, and Maryland employed a weighted lottery to allocate more than 10,000 individuals to COVID-19 monoclonal antibody treatment.^35,36^ Despite other countries experiencing critical shortages of ventilators^37^ and COVID-19 medications, we find little evidence of lotteries being used outside the United States. It is notable, therefore, that the United States had the highest agreement with lottery being an appropriate allocation mechanism of any developed country in our study. This may be due to the use of lotteries in other contexts (e.g., immigration lottery) or may simply reflect underlying support for lottery allocation among the US population. In a global context, there are only 2 countries with higher agreement with lottery (China and India), and the US results are in stark contrast with many comparable developed countries, mainly in Europe. Beyond country variation response, heterogeneity was associated with a number of characteristics, with generally greater support for lottery allocation by males, those on higher income, those with the very lowest levels of education, and those with a right-leaning ideology.

Although there are differences in methodology, the wide variation in preferences for lottery allocation between and within countries accords with the findings of Awad et al.,^26^ who conducted a multicountry study to look at the allocation of scarce ventilators in hospitals. All participants received the same survey and were asked to rate the usability of 5 triage metrics and 2 no-triage mechanisms (including random lottery) to allocate ventilators. They found that, across all countries, public preferences were polarized, with people either preferring methods involving no triage (lotteries or first-come, first-served) or extensive triage using all available triage criteria. Similarly, among a wide variety of different allocation principles, Lee et al.^38^ found the least support for the use of random selection, based on a survey in Korea. In a study in Portugal, lotteries were considered the least fair mechanism, and highest priority was given to the prognosis and severity of health condition.^39^

While random allocation is rarely used as a health care–rationing mechanism, it is, albeit for entirely different reasons, routinely used in clinical research, in the context of enrollment in randomized clinical trials. A research question that arises from our study is whether there is also substantive variation in the acceptance of being enrolled in clinical trials. Given the importance of the rate and diversity of recruitment,^40,41^ this topic warrants further attention.

### Strengths and Limitations

A key strength of the study is its use of a consistent, harmonized survey across representative samples of a wide range of countries and diverse social and economic contexts. The study by Awad et al.^26^ is the only related work we are aware of that surveyed more countries, but only 4 of those countries used representative samples, and our overall samples are larger. Another key strength is our use of randomization, which ensures that responses to agreement with lottery allocation are not contaminated by prior questions about agreement with allocation by expert committee or vice versa.

This study also has several limitations. The first is that it relied on an opt-in panel, whereas probability-based panels have been found to be more accurate.^42^ Although opt-in panels are more prone to errors, this should not compromise our analysis of the comparative agreement for expert committee and lottery allocation. Beyond our use of quota sampling, which should avoid some of these concerns, our experimental design also ensures the validity of our conclusions because respondents were randomly assigned to either the lottery or expert committee vignette. Consequently, any errors introduced by the opt-in panel are likely to be randomly distributed across the 2 groups. This random assignment ensures that, on average, the impact of these errors is the same for both allocation methods, preserving the validity of the comparative findings on the appropriateness of these allocation mechanisms.

Next, the survey-based experiment analyzed in this study was part of the larger CANDOUR study,^27^ and therefore, we are unable to rule out the possibility that previous questions may have contaminated the results of the agreement with the appropriateness of each allocation mechanism. While no other questions on medical resource allocation were asked as part of the survey, it is still possible that other questions related to vaccines and COVID-19 may have affected responses. However, as outlined above, the random assignment of vignettes ensures that, on average, these errors would be the same for each allocation mechanism, maintaining the validity of our comparative findings.

Another limitation is that our study focuses solely on 2 allocation mechanisms, without exploring more complex variations or combined approaches. For instance, our vignettes specifically examine vaccine allocation among nurses, comparing a within-group lottery-based system, similar to that suggested by the US National Academy of Sciences’ report,^16^ with allocation by expert committee. However, numerous alternative allocation mechanisms could have been considered. In addition, the study uses only a single vignette about vaccine allocation among nurses. While this example was salient at the time, the contexts in which lotteries or other mechanisms might be applied are numerous and diverse. Our approach prioritized simplicity, asking respondents to evaluate a single, specific, and simple scenario that we believe would provide more accurate responses due to the reduced cognitive load.

Relatedly, it is possible that the wording of the expert committee allocation vignette may have implied that the expert committee was able to precisely determine which nurses would have benefitted most from receiving the vaccine. As discussed in the introduction, such allocation decisions can be challenging, especially given the large number of nurses included in our vignettes. Given inherent uncertainties and the cognitive limits of physicians, it may not be possible to deterministically allocate vaccines to the nurses who would benefit most from the vaccine. It is possible that if respondents were made aware of the challenges associated, then the differences in agreement with allocation by expert committee and lottery may have been reduced.

It is also possible that our estimates of the agreement with the appropriateness of lottery allocation are not fully representative of the population. It is possible that our sampling strategy may have resulted in a sample of respondents that does not accurately reflect the broader population. This issue is especially relevant given that we used a Web-based survey in low-income countries, where internet access may be unevenly distributed. To address these potential biases, we used quota sampling and poststratification weighting. With the exception of India and Uganda, which were primarily samples of urban communities, these methods ensured that the national samples were representative of the target populations with respect to key demographic variables. However, because our data are derived from a sample rather than the entire population, we cannot entirely rule out the possibility that our estimates do not perfectly reflect the population-level agreement with allocation using a lottery.

Finally, our study does not account for the broader implications of allocation mechanisms on vaccine uptake. Research by Bruine de Bruin et al.^43^ suggests that individuals who are not allocated vaccine eligibility may be less inclined to accept the vaccine when eligibility is granted later on. This effect was found to vary depending on the allocation mechanism used. Overall, Bruine de Bruin et al. found that when individuals were initially overlooked for vaccination, the probability of refusal was 22% higher when vaccines were allocated by lottery compared with when the allocation mechanism was unspecified. However, refusal rates were even higher when the initial allocation strategy prioritized “high-risk individuals living in group housing,”“high-risk individuals living with their families,” and “people who live in areas with more COVID-19 infections” than when allocation was determined by lottery. This may suggest that the perceived fairness and transparency of lottery-based allocation can lead to less negative public reaction than need-based mechanisms. Therefore, beyond evaluating the agreement with the appropriateness of these approaches, policy makers must also consider the downstream effects of their choice of allocation mechanism, which was not addressed in this study.

## Conclusion

In the context of scarce medical resources and their allocation among people of equal need, the medical ethics literature has made compelling arguments in favor of lotteries. It has been argued, for instance, that lotteries are fairer than either a first-come, first-served allocation or allocation based on clinician referral,^35^ both of which have been deemed more likely to exacerbate existing health disparities than to mitigate them. Weighted lotteries in particular, in which the chance of being allocated is randomized, but with a higher probability for more deprived individuals, hold promise for allocating scarce medical resources in a way that actively tackles existing health disparities. It is in circumstances such as these that we see the most compelling case for potential greater use of random allocation of medical resources. However, it is worth noting that lotteries may even have some potential advantages when allocating scarce medical resources among those with unequal needs: 1) compared with lottery allocation, which can be administered at relatively low cost, medical experts’ time is costly and is a drain on potential resources, and 2) it has been documented that medical experts are biased (both unconsciously and sometimes consciously) when determining treatment.^44–46^ Future work may seek to evaluate the receptiveness of various populations to some of these arguments and to find effective ways to communicate the potential advantages of lotteries in certain contexts.

## Supplemental Material

sj-docx-1-mdm-10.1177_0272989X251367777 – Supplemental material for Lottery or Triage? Controlled Experimental Evidence from the COVID-19 Pandemic on Public Preferences for Allocation of Scarce Medical Resources Supplemental material, sj-docx-1-mdm-10.1177_0272989X251367777 for Lottery or Triage? Controlled Experimental Evidence from the COVID-19 Pandemic on Public Preferences for Allocation of Scarce Medical Resources by Rhys Llewellyn Thomas, Laurence SJ Roope, Raymond Duch, Thomas Robinson, Alexei Zakharov and Philip Clarke in Medical Decision Making
