# Supplementary material for: Lottery or Triage? Controlled Experimental Evidence from the COVID-19 Pandemic on Public Preferences for Allocation of Scarce Medical Resources
Source: Med Decis Making. 2025 Sep 27;46(1):102–15. doi: 10.1177/0272989X251367777 (PMC12705886; doi:10.1177/0272989X251367777)
Supplement: sj-docx-1-mdm-10.1177_0272989X251367777 – Supplemental material for Lottery or Triage? Controlled Experimental Evidence from the COVID-19 Pandemic on Public Preferences for Allocation of Scarce Medical Resources [file sj-docx-1-mdm-10.1177_0272989X251367777.docx]

**Supplementary Materials**

**Table A1. Characteristics of Participants using Sample Weights**

|  |  | | |  |
| --- | --- | --- | --- | --- |
|  | **Expert Committee** | **Lottery** | **P-value**  **Test for differences** | |
|  |  |  |  | |
| **Age** | 44.336 (16.578) | 44.838 (16.039) | 0.490 | |
|  |  |  |  | |
| **Age Categories** |  |  |  | |
| Age: 18−39 | 2,915 (43.1%) | 3,037 (41.4%) | 0.512 | |
| Age: 40-60 | 2,280 (33.7%) | 2,421 (33.0%) |  | |
| Age: 60+ | 1,550 (22.9%) | 1,871 (25.5%) |  | |
| Age: Missing/ Out of range | 12 (0.2%) | 10 (0.1%) |  | |
|  |  |  |  | |
| **Gender** |  |  |  | |
| Gender: Female | 3,608 (53.4%) | 3,796 (51.7%) | 0.467 | |
| Gender: Male | 3,144 (46.5%) | 3,540 (48.2%) |  | |
| Gender: Other | 1 (0.0%) | 2 (0.0%) |  | |
| Gender: Missing | 3 (0.1%) | 1 (0.0%) |  | |
|  |  |  |  | |
| **Political Ideology** |  |  |  | |
| Ideology: Left | 709 (10.5%) | 755 (10.3%) | 0.996 | |
| Ideology: Centre | 3,147 (46.6%) | 3,424 (46.7%) |  | |
| Ideology: Right | 1,058 (15.7%) | 1,145 (15.6%) |  | |
| Ideology: Missing | 1,843 (27.3%) | 2,015 (27.5%) |  | |
|  |  |  |  | |
| **Income Category** |  |  |  | |
| Income: High | 3,772 (56.5%) | 4,037 (55.6%) | 0.343 | |
| Income: Low | 2,235 (33.5%) | 2,312 (31.9%) |  | |
| Income: Missing | 669 (10.0%) | 910 (12.5%) |  | |
|  |  |  |  | |
| **Education** |  |  |  | |
| Education: Primary | 1,807 (26.7%) | 2,302 (31.4%) | 0.054 | |
| Education: Secondary | 2,983 (44.2%) | 3,163 (43.1%) |  | |
| Education: University | 1,963 (29.1%) | 1,870 (25.5%) |  | |
| Education: Missing | 3 (0.0%) | 3 (0.0%) |  | |

**Table A2. Agreement with allocation by lottery versus expert committee**

|  | **Expert committee** | | **Lottery** | | **Expert committee Mean – Lottery Mean (95% CI)** |
| --- | --- | --- | --- | --- | --- |
| **Characteristic** | **N** | **Mean (95% CI)** | **N** | **Mean (95% CI)** |  |
|  |  |  |  |  |  |
| **Estimated Using Survey Weights** | | |  |  |  |
| **Overall** | 7668 | 61.19 (60.04 - 62.35) | 7712 | 37.25 (34.86 - 39.65) | 23.94 (21.27 - 26.61) |
|  |  |  |  |  |  |
| **Country** |  |  |  |  |  |
| Australia | 588 | 63.85 (61.21 - 66.48) | 634 | 43.75 (41.01 - 46.5) | 20.09 (16.3 - 23.89) |
| Brazil | 626 | 59.21 (55.88 - 62.55) | 670 | 34.65 (31.25 - 38.06) | 24.56 (19.81 - 29.32) |
| Canada | 509 | 62.21 (59.3 - 65.12) | 536 | 40.52 (37.39 - 43.66) | 21.69 (17.42 - 25.96) |
| Chile | 508 | 51.25 (45.44 - 57.06) | 484 | 21.1 (15.07 - 27.13) | 30.15 (21.8 - 38.5) |
| China | 617 | 69.49 (65.61 - 73.37) | 630 | 60.54 (55.41 - 65.66) | 8.96 (2.54 - 15.37) |
| Colombia | 615 | 58.47 (54.48 - 62.47) | 557 | 28.85 (24.69 - 33.02) | 29.62 (23.86 - 35.38) |
| France | 489 | 53.97 (50.94 - 56.99) | 502 | 22.24 (19.42 - 25.06) | 31.73 (27.61 - 35.85) |
| India | 580 | 69.77 (64.67 - 74.87) | 538 | 62.33 (54.45 - 70.21) | 7.44 (-1.92 - 16.8) |
| Italy | 483 | 61.3 (58.13 - 64.46) | 489 | 33.85 (30.4 - 37.3) | 27.45 (22.78 - 32.12) |
| Russia | 583 | 56.97 (54.31 - 59.62) | 609 | 32.33 (29.61 - 35.05) | 24.63 (20.84 - 28.43) |
| Spain | 563 | 61.13 (58.67 - 63.58) | 510 | 28.93 (26.26 - 31.6) | 32.19 (28.57 - 35.81) |
| Uganda | 506 | 58.73 (47.4 - 70.06) | 521 | 29.15 (8.83 - 49.48) | 29.58 (6.38 - 52.79) |
| UK | 512 | 65.82 (63.22 - 68.42) | 534 | 34.47 (31.76 - 37.17) | 31.35 (27.61 - 35.09) |
| US | 489 | 63.62 (61.2 - 66.03) | 498 | 49.6 (46.67 - 52.52) | 14.02 (10.24 - 17.8) |
|  |  |  |  |  |  |
| **Estimated without Survey Weights** | | |  |  |  |
| **Gender** |  |  |  |  |  |
| Female | 3683 | 60.76 (59.81 - 61.71) | 3681 | 35.02 (33.96 - 36.08) | 25.74 (24.32 - 27.16) |
| Male | 3945 | 64.34 (63.45 - 65.24) | 3986 | 41.59 (40.53 - 42.65) | 22.76 (21.37 - 24.14) |
|  |  |  |  |  |  |
| **Age** |  |  |  |  |  |
| Age: 18-39 | 3657 | 62.86 (61.96 - 63.76) | 3684 | 39.21 (38.13 - 40.28) | 23.65 (22.25 - 25.05) |
| Age: 40-59 | 2532 | 62.83 (61.67 - 63.99) | 2513 | 39.15 (37.8 - 40.49) | 23.69 (21.91 - 25.46) |
| Age: 60+ | 1471 | 61.37 (59.81 - 62.92) | 1510 | 35.3 (33.64 - 36.97) | 26.07 (23.79 - 28.34) |
|  |  |  |  |  |  |
| **Income** |  |  |  |  |  |
| Income: High | 3807 | 61.37 (60.44 - 62.29) | 3908 | 36.14 (35.11 - 37.17) | 25.23 (23.84 - 26.61) |
| Income: Low | 2888 | 65.46 (64.41 - 66.51) | 2822 | 42.65 (41.37 - 43.93) | 22.81 (21.16 - 24.46) |
|  |  |  |  |  |  |
| **Education** |  |  |  |  |  |
| Primary Completed | 882 | 64.76 (62.81 - 66.71) | 918 | 44.33 (42.09 - 46.58) | 20.43 (17.46 - 23.4) |
| Secondary Completed | 2980 | 59.71 (58.64 - 60.78) | 3129 | 35.49 (34.34 - 36.64) | 24.22 (22.65 - 25.79) |
| University Completed | 3671 | 64.59 (63.69 - 65.5) | 3562 | 39.58 (38.48 - 40.69) | 25.01 (23.58 - 26.44) |
|  |  |  |  |  |  |
| **Political Ideology** |  |  |  |  |  |
| Centre | 3573 | 61.86 (60.96 - 62.76) | 3557 | 35.95 (34.93 - 36.96) | 25.91 (24.55 - 27.27) |
| Left | 865 | 61.62 (59.55 - 63.7) | 847 | 28.24 (26.13 - 30.35) | 33.38 (30.43 - 36.34) |
| Right | 1301 | 69.59 (67.97 - 71.2) | 1260 | 50.01 (47.89 - 52.14) | 19.57 (16.91 - 22.24) |

The table shows the mean agreement and 95% confidence intervals for the lottery and expert committee vignettes. Mean agreement is presented overall and by observable characteristics. Additionally, the table displays the mean differences between agreement with the appropriateness of allocation by expert committee and lottery, and the corresponding 95% confidence interval, calculated using OLS.

**Table A3. Association between respondent characteristic and agreement with the appropriateness of the allocation mechanism**

|  | **Expert committee allocation agreement** | | | **Lottery allocation agreement** | | | |  | |
| --- | --- | --- | --- | --- | --- | --- | --- | --- | --- |
| Variable | Coefficient Estimate | [CI 95%] | | | Coefficient Estimate | | [CI 95%] | |  |
|  |  |  | | |  | |  | |  |
| **Gender** |  |  | | |  | |  | |  |
| Female | -1.01 | [-2.96,0.95] | | | -3.01 | | [-6.39,0.38] | |  |
| Male | Reference Category | |  | | |  |  |  |  |
| Other | -5.63** | [-9.53,-1.73] | | | -33.09*** | | [-42.80,-23.38] | |  |
| Missing | -28.00*** | [-43.30,-12.70] | | | 18.75*** | | [8.81,28.69] | |  |
|  |  | |  | | |  |  |  |  |
| **Age** |  | |  | | |  |  |  |  |
| 18-39 | Reference Category | |  | | |  |  |  |  |
| 40-60 | 2.90* | [0.12,5.68] | | | 0.25 | | [-3.41,3.91] | |  |
| 60+ | 1.15 | [-1.95,4.25] | | | -0.68 | | [-5.77,4.41] | |  |
| Missing/ Out of range | -6.56 | [-42.22,29.11] | | | 2.91 | | [-34.06,39.87] | |  |
|  |  |  | | |  | |  | |  |
| **Income** |  |  | | |  | |  | |  |
| High | -1.04 | [-3.50,1.42] | | | -6.35*** | | [-9.83,-2.88] | |  |
| Low | Reference Category | |  | | |  |  |  |  |
| Missing | -4.09 | [-8.52,0.34] | | | -8.76 | | [-18.92,1.41] | |  |
|  |  | |  | | |  |  |  |  |
| **Education** |  | |  | | |  |  |  |  |
| Primary | Reference Category | |  | | |  |  |  |  |
| Secondary | 0.13 | [-3.43,3.69] | | | 4.88 | | [-1.22,10.97] | |  |
| University | 5.76** | [1.78,9.75] | | | 2.91 | | [-1.52,7.35] | |  |
| Missing | 23.33 | [-0.64,47.29] | | | 2.29 | | [-16.74,21.32] | |  |
|  |  |  | | |  | |  | |  |
| **Political Ideology** |  |  | | |  | |  | |  |
| Left | 0.88 | [-2.95,4.70] | | | 0.83 | | [-10.93,12.58] | |  |
| Centre | Reference Category | |  | | |  |  |  |  |
| Right | 6.59*** | [3.70,9.49] | | | 11.31*** | | [6.06,16.55] | |  |
| Missing | -3.43 | [-7.92,1.07] | | | -4.15* | | [-8.00,-0.30] | |  |
|  |  |  | | |  | |  | |  |
| **Country** |  |  | | |  | |  | |  |
| Australia | 3.24 | [-0.29,6.77] | | | -2.49 | | [-6.39,1.41] | |  |
| Brazil | -0.78 | [-4.95,3.39] | | | -11.75*** | | [-16.49,-7.02] | |  |
| Canada | 2.06 | [-1.67,5.79] | | | -5.38* | | [-9.76,-0.99] | |  |
| Chile | -8.74** | [-14.87,-2.62] | | | -24.03*** | | [-29.62,-18.44] | |  |
| China | 14.51*** | [8.13,20.89] | | | 21.18*** | | [13.50,28.87] | |  |
| Colombia | -1.56 | [-6.23,3.12] | | | -15.64*** | | [-20.85,-10.43] | |  |
| France | -7.97*** | [-11.90,-4.04] | | | -23.31*** | | [-27.38,-19.23] | |  |
| India | 8.53** | [3.01,14.04] | | | 13.91** | | [5.08,22.74] | |  |
| Italy | 1.1 | [-2.89,5.10] | | | -12.76*** | | [-17.49,-8.04] | |  |
| Russia | 0.23 | [-5.20,5.66] | | | -9.61*** | | [-14.88,-4.34] | |  |
| Spain | -0.11 | [-3.70,3.48] | | | -16.34*** | | [-21.10,-11.57] | |  |
| Uganda | 2.73 | [-8.71,14.17] | | | -14.79 | | [-32.16,2.59] | |  |
| UK | 5.31** | [1.76,8.86] | | | -12.08*** | | [-16.11,-8.05] | |  |
| US | Reference Category | |  | | |  |  |  |  |
|  |  |  | | |  | |  | |  |
| Constant | 58.16*** | [52.63,63.69] | | | 47.82*** | | [41.26,54.38] | |  |
| N |  | | |  | | | |  | |
|  | 7440 | | | 7459 | | | |  |  |

Table shows OLS regression estimates for the association between respondent characteristics and agreement with the appropriateness of each allocation mechanism. Agreement with expert committee allocation and lottery allocation are separately estimated. Estimates do not use weights.

*** denotes P-value of 0.01 or less ,** denotes P-value of 0.05 or less,* denotes P-value of 0.10 or less.
